# An NFC on Two-Coil WPT Link for Implantable Biomedical Sensors under Ultra-Weak Coupling

**DOI:** 10.3390/s17061358

**Published:** 2017-06-11

**Authors:** Chen Gong, Dake Liu, Zhidong Miao, Wei Wang, Min Li

**Affiliations:** Institute of Application Specific Instruction-Set Processors, Beijing Institute of Technology, 5 South Zhongguancun Street, Haidian District, Beijing 100081, China; gongchen@bit.edu.cn (C.G.); zhidongmiao@bit.edu.cn (Z.M.); 3120130295@bit.edu.cn (W.W.); limin2015@bit.edu.cn (M.L.)

**Keywords:** biomedical telemetry, near-field communication, wireless power transmission, inductive link, implantable biomedical sensors

## Abstract

The inductive link is widely used in implantable biomedical sensor systems to achieve near-field communication (NFC) and wireless power transfer (WPT). However, it is tough to achieve reliable NFC on an inductive WPT link when the coupling coefficient is ultra-low (0.01 typically), since the NFC signal (especially for the uplink from the in-body part to the out-body part) could be too weak to be detected. Traditional load shift keying (LSK) requires strong coupling to pass the load modulation information to the power source. Instead of using LSK, we propose a dual-carrier NFC scheme for the weak-coupled inductive link; using binary phase shift keying (BPSK) modulation, its downlink data are modulated on the power carrier (2 MHz), while its uplink data are modulated on another carrier (125 kHz). The two carriers are transferred through the same coil pair. To overcome the strong interference of the power carrier, dedicated circuits are introduced. In addition, to minimize the power transfer efficiency decrease caused by adding NFC, we optimize the inductive link circuit parameters and approach the receiver sensitivity limit. In the prototype experiments, even though the coupling coefficient is as low as 0.008, the in-body transmitter costs only 0.61 mW power carrying 10 kbps of data, and achieves a 1 × 10${}^{-7}$ bit error rate under the strong interference of WPT. This dual-carrier NFC scheme could be useful for small-sized implantable biomedical sensor applications.

## 1. Introduction

Implantable battery-less biomedical sensor systems will play an important role in future medical diagnose and treatment [1,2]. The simultaneous power and data transmission is a fundamental requirement for the implantable sensor systems, and it should be wirelessly powered continuously when it is working. The inductive link consisting of a magnetic-coupled coil pair is a viable method to transmit wireless power [3] and data [4]. However, the application requirements of the implantable sensor devices often set strict limits on the size and number of the coils, thus leading to the ultra-weak coupling between the coils. The ultra-weak coupling challenges the traditional inductive link in transmitting stable power and reliable data. The weak coupling has become a major constraint for miniaturizing the size of sensor devices.

Traditionally, the technique of radio frequency identification devices (RFID) is widely used in implantable biomedical sensor systems [5,6,7]. For the uplink, RFID characteristically uses load-shift keying (LSK). Thus, RFID could transmit power and uplink data simultaneously through only one coil pair and one carrier. However, LSK generally can only be reliable on the coupling (*k*= 0.5∼0.1). According to our review of LSK publications, when the coupling efficient is less than 0.1, the bit error rate (BER) of LSK remarkably increases, and when the coupling efficient is less than 0.05, the current variants induced by the load modulation can hardly be detected. For the downlink, RFID adopts amplitude shift keying (ASK) on the power carrier for the simplicity of the demodulation. However, ASK has to alter the power carrier amplitude remarkably, thus reducing the utility of the wireless power transmission (WPT). To improve the power stability and efficiency, researchers try to use frequency shift keying (FSK), binary phase shift keying (BPSK), differential phase shift keying (DPSK) and offset quadrature shift keying (OQPSK) [8,9,10,11] for the downlink.

Furthermore, the situation of an ultra-low coupling coefficient (0.01 typically) has seldom been studied for the simultaneous power and data transmission.

**Figure 1:**
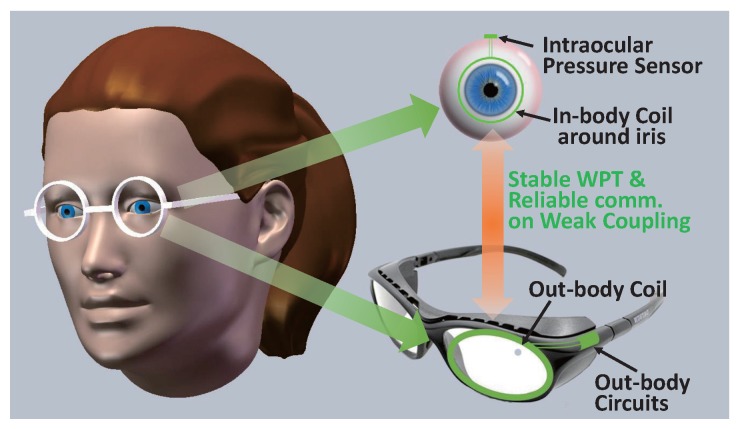
Inductive wireless link of the implantable sensor system for glaucoma treatment.

In this paper, we propose a near-field communication (NFC) attached to an inductive WPT link, tailored to an implantable sensor system for glaucoma treatment. The intraocular sensor device will be implanted in patients’ eyeball to monitor and regulate the intraocular pressure (IOP), as seen Figure 1. The IOP is measured by a micro-hydrostatic sensor. The uplink sends the data of the pressure for monitoring the IOP and the data of the peak voltage on the load for adaptively adjusting the transmitted power of WPT. When the measured IOP is above the normal IOP, the downlink sends the commands to control an actuator (micro-pump) to regulate the IOP. High IOP is considered a leading risk factor for glaucoma, and lowering IOP continues to be the only evidence-based treatment to prevent its development or to reduce the progression rate [12,13]. Due to the limits of the weight and size in the eyeball, the battery cannot be integrated, and only one coil could be implanted for NFC and WPT. In a word, the wireless link of this device needs to transmit power and data simultaneously through the same coil pair under weak coupling.

The proposed NFC uses two carriers through the same coil pair. One carrier is the power carrier for the power and downlink data transmission, while the other is the uplink carrier for the uplink data transmission. BPSK modulation is used in the downlink to minimize the amplitude ripple of the power carrier. BPSK is also utilized in the uplink to be able to demodulate the weak signal under strong interference. In the inductive link circuit, dedicated passive components are introduced and optimized to minimize the power transfer efficiency (PTE) decrease caused by adding the data links. For the uplink, we firstly explore the constraint of avoiding the out-body receiver from being saturated by the power carrier and the limit of uplink receiver sensitivity. Then, we optimize the circuit parameters to get the maximal PTE and at the same time keep the received uplink signal power to be sufficient for reliable demodulation. For the downlink, a shaping filter is introduced in the downlink modulator to reduce the impact of BPSK modulation on the WPT power amplifier (Class E PA). Finally, a prototype was implemented. Our proposed NFC overcomes the strong interference from the power carrier and achieves reliable communication under ultra-weak coupling. At the same time, our NFC causes less PTE loss and negligible amplitude ripple on the WPT power carrier, compared with the traditional NFC using LSK.

This paper focuses on the design and optimization of the physical link layer, as it is the key to achieve the simultaneous power and data transmission. The rest of this paper is arranged as follows. Section 2 presents the overview to introduce the system structure and considerations of this dual-carrier NFC scheme. Section 3 presents the equivalent model and analysis of the inductive link circuit. Section 4 discusses the minimization of the interactions between the NFC and the WPT. Section 5 presents the prototype implementation and the measured results. Section 6 is the conclusion.

## 2. Dual-Carrier NFC System

### 2.1. System Overview

**Figure 2:**
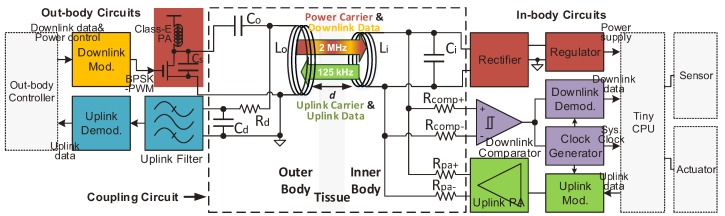
Block diagram of our implantable biomedical sensor system utilizing the proposed inductive link. The WPT and the NFC share the same coil pair by using two carriers. This paper focuses on analyzing and optimizing the inductive link circuit, which is enclosed in the dashed box.

The system block diagram of our implantable sensor system is shown in Figure 2. The WPT and the NFC share the same coil pair through dual carriers.

In the power link (the dark red blocks), the out-body coil ($L_{o}$) is driven by a Class E power amplifier (PA) to generate the power carrier [14,15]. The Class E topology is chosen for its high efficiency, which is theoretically 100%. A high-speed power MOSFET is turned on and off at 2-MHz frequency, with its switched-on period controlled dedicatedly to ensure the Class E to operate at its optimal point for high power efficiency. A rectifier and a regulator qualify the harvested power carrier from the in-body coil ($L_{i}$) to supply the in-body circuit. The pulse-width modulation (PWM) on the power carrier is used for the adaptive power adjustment of the power link. The adaptive power adjustment adjusts the transmitted power of the Class E PA to keep the PDL constant over different coupling coefficients in a wide operating range of coil separation. That is, when the coupling coefficient increases, the transmitted power of the Class E PA will be reduced adaptively.

The downlink modulator (the golden yellow block) performs the BPSK modulation on the power carrier. The in-body receiver (the purple grey blocks) acquires the modulated power carrier from $L_{i}$. In the in-body receiver, a comparator shapes the modulated power carrier to a square-wave signal and feeds it to the clock generator and the downlink demodulator. The clock generator offers high quality clocks by tracking the power carrier frequency during the period when the power carrier is not modulated [16]. The downlink demodulator is an all-digital BPSK demodulator with ultra-low power [17].

The in-body transmitter (the green blocks) generates the BPSK-modulated uplink carrier and drives $L_{i}$ by the uplink PA. The uplink modulator is simply implemented by a multiplexer to select the carrier or the inverted carrier. The out-body receiver (the cyan blocks) acquires the uplink signal from $L_{o}$. Because the power carrier brings strong interference to the out-body receiver, the high-order active filter (uplink filter) is installed before the uplink demodulator [18].

The uplink and the downlink work in half-duplex to avoid their mutual interference. The data rate of the uplink and the downlink are both 10 kbps in our system, as it is a sufficient data rate to send intraocular pressure data, in-body system power statuses and control commands.

### 2.2. Inductive Link Circuit

As seen in Figure 2, $L_{o}$ and $L_{i}$ both resonate at the power carrier frequency ($f_{p}$) with their tuning capacitors $C_{o}$ and $C_{i}$. The critical coupling factor for our system is 0.11 according to [19]. As the operating range of our system is 0.1 > *k* > 0.01, our system always works in the under-coupled situation (the coupling coefficient is below the critical coupling coefficient). Therefore, the out-body coil and the in-body coil both resonate at the power carrier frequency with their tuning capacitors to maximize the power transfer efficiency. $R_{d}$ and $C_{d}$ constitute an RCdivider (a low-pass filter) to prevent the uplink filter in the out-body receiver from being saturated by the power carrier interference. High impedance probe resistors (HIPRs) $R_{d}$, $R_{\mathrm{comp}+}$, $R_{\mathrm{comp}-}$, $R_{\mathrm{pa}+}$ and $R_{\mathrm{pa}-}$ are added between the coils and the ports of the transceivers, to isolate the NFC from the WPT.

### 2.3. Dual Carriers

The power carrier operates at 2 MHz ($f_{p}$) in consideration of the less energy loss upon biological tissues [20]. The downlink data are modulated on the power carrier. The downlink uses BPSK instead of ASK, which is widely used in RFID, because ASK reduces PTE due to the unavoidable carrier amplitude variation.

The uplink data are modulated on the uplink carrier by using BPSK. The uplink carrier operates at 125 kHz ($f_{\mathrm{ul}}$). It is $\frac{1}{16}$-times lower than $f_{P}$ and offers sufficient transition band span to simplify the out-body receiving filter. The lower frequency of the uplink carrier also helps to keep away from the interferences of the power carrier harmonics.

Since the uplink carrier is transferred through the inductive link resonated at 2 MHz, the 125-kHz uplink signal could be attenuated seriously through this link. In order to guarantee the reliable uplink communication, the minimum receiving signal power of the uplink is analyzed and guaranteed to be sufficient for the reliable demodulation in our operating range. The details will be presented in Section 4.

### 2.4. Application Requirements on Coils

In the intraocular sensor device, the coil sizes are limited by the application requirements and are decided by the doctors from our cooperative hospital. To avoid blocking the eyesight, the in-body coil is designed to surround the periphery of the cornea hidden behind the eyelid. The out-body coil will be embedded inside the glasses frame in front and around the eye that suffers from glaucoma. Table 1 lists the parameters of the coils we use. The coil parameters are optimized for a higher PTE with sufficient power delivered to load (PDL) under the strict constraints on their size in [21].

The separation (*d*) between the coils could vary from 10 mm to 40 mm due to the variations of the patient facial forms. Accordingly, the coupling coefficient (*k*) varies from 0.1 to 0.01 typically in our sensor system, which is considered as the operating range of our link.

## 3. Inductive Link Circuit Modeling

### 3.1. Equivalent Circuit Simplification

**Figure 3:**
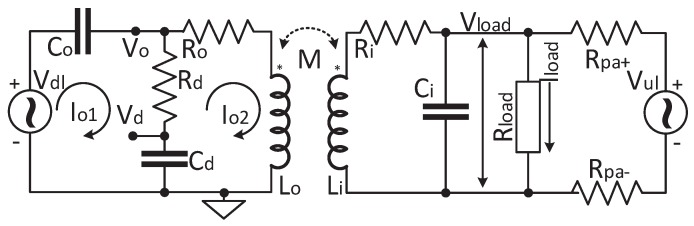
Circuit topology of the proposed inductive wireless link.

Figure 3 shows the equivalent circuit topology of the inductive link circuit. The Class E PA and the uplink PA are simplified as ideal AC voltage sources. $V_{\mathrm{dl}}$ is the ideal AC voltage source of the power carrier. The internal resistance of $V_{\mathrm{dl}}$ is omitted, because the internal impedance of the Class E PA has negligible direct impact on the inductive link circuit optimization. Similarly, $V_{\mathrm{ul}}$ is the equivalent AC voltage source of the uplink carrier, while its internal resistance is also omitted because its internal resistance can be regard as a part of $R_{\mathrm{pa}+}$ and $R_{\mathrm{pa}-}$. $R_{o}$ and $R_{i}$ are the equivalent parasitic resistance of the out-body coil and the in-body coil, respectively. $L_{o}$ and $L_{i}$ are the inductances of the out-body coil and the in-body coil, respectively. $R_{\mathrm{load}}$ is the equivalent resistor of the power load. In this circuit topology, $R_{\mathrm{load}}$ is represented by a resistor whose value is about 2 kΩ according to our design. $R_{\mathrm{comp}+}$ and $R_{\mathrm{comp}-}$ are omitted in the circuit topology because the comparator input impedance is sufficiently high.

### 3.2. Power Link (Downlink) Transfer Functions

When analyzing the power link in Figure 3, the source of the uplink can be considered as a short circuit. By leveraging the mesh-current technique [22], the voltage and current transfer functions of the power link are derived as shown in Equations (1) and (2), where $M=k\sqrt{L_{o}L_{i}}$ is the mutual inductance between the coils and *s* is the Laplacian. Equations (1) and (2) will be used to calculate the PTE in Section 4.1.3. The relation between $V_{o}\left(s\right)$ and $V_{\mathrm{load}}\left(s\right)$ is denoted in Equation (3). Equation (3) will be used to calculate the power carrier amplitude on the out-body coil in Section 4.1.1.

(1) $$\left\{\begin{array}{c} \frac{V_{\mathrm{load}}\left(s\right)}{V_{\mathrm{dl}}\left(s\right)}=\frac{sM}{\left(\frac{1}{Z_{i}}\left(sL_{i}+R_{i}\right)+1\right)\left(\left(sL_{o}+R_{o}\right)\left(1+\frac{1}{sC_{o}Z_{d}}\right)+\frac{1}{sC_{o}}\right)-s^{2}M^{2}\frac{1}{Z_{i}}\left(1+\frac{1}{sC_{o}Z_{d}}\right)}\\ Z_{i}=R_{\mathrm{load}}\left|\right|\frac{1}{sC_{i}}\left|\right|\left(R_{\mathrm{pa}+}+R_{\mathrm{pa}-}\right);Z_{d}=\frac{1}{sC_{d}}+R_{d} \end{array}\right.,$$

(2) $$\frac{I_{\mathrm{load}}\left(s\right)}{I_{o1}\left(s\right)}=\frac{Z_{d}}{Z_{d}+sL_{o}+R_{o}-\frac{s^{2}M^{2}}{sL_{i}+R_{i}+Z_{i}}}\cdot \frac{sM}{sL_{i}+R_{i}+Z_{i}}\cdot \frac{Z_{i}}{R_{\mathrm{load}}},$$

(3) $$\frac{V_{\mathrm{load}}\left(s\right)}{V_{o}\left(s\right)}=\frac{sMZ_{i}}{\left(sL_{i}+R_{i}+Z_{i}\right)\left(sL_{o}+R_{o}-\frac{s^{2}M^{2}}{sL_{i}+R_{i}+Z_{i}}\right)}.$$

### 3.3. Uplink Transfer Function

Similarly, considering $V_{\mathrm{dl}}$ as a short circuit in Figure 3, the voltage transfer function of the uplink is derived, as shown in Equation (4). It denotes the relation between $V_{d}\left(s\right)$ and $V_{\mathrm{ul}}\left(s\right)$ and will be used to calculate the received uplink signal power in Section 4.1.3.

(4) $$\left\{\begin{array}{c} \frac{V_{d}\left(s\right)}{V_{\mathrm{ul}}\left(s\right)}=\frac{1}{1+sR_{d}C_{d}}\cdot \frac{1}{1+\frac{1}{Z_{\mathrm{load}}}\left(R_{\mathrm{pa}+}+R_{\mathrm{pa}-}\right)}\cdot \frac{sM}{\left(\frac{1}{Z_{o}}\left(R_{o}+sL_{o}\right)+1\right)\left(sL_{i}+R_{i}+Z_{\mathrm{load}}\right)-\frac{1}{Z_{o}}s^{2}M^{2}}\\ Z_{o}=\frac{1}{sC_{o}}\left|\right|\left(R_{d}+\frac{1}{sC_{d}}\right);Z_{\mathrm{load}}=R_{\mathrm{load}}\left|\right|\frac{1}{sC_{i}} \end{array}\right..$$

### 3.4. Signal-To-Interference Ratio of the Uplink

The power carrier is the major interference to the out-body uplink receiver. We evaluate the impact of the power carrier interference through calculating the signal-to-interference ratio (SIR) at $V_{d}$ (the output of the RC divider). The SIR is defined in Equation (5), where $V_{d}\left(j\omega_{\mathrm{ul}}\right)$ is calculated based on the uplink transfer function of $\frac{V_{d}\left(s\right)}{V_{\mathrm{ul}}\left(s\right)}$ in Equation (4), and the divide ratio of the RC divider $\frac{1}{1+sR_{d}C_{d}}$; $V_{d}\left(j\omega_{\mathrm{dl}}\right)$ is calculated based on the transfer function of $\frac{V_{\mathrm{load}}\left(s\right)}{V_{o}\left(s\right)}$ in Equation (3) and the divide ratio of the RC divider $\frac{1}{1+sR_{d}C_{d}}$. When *k* is the minimum value (0.01) in the operating range, the SIR is about −100 dB, which is hard to demodulate directly. Thus, the uplink receiving filter is introduced in the out-body receiver to improve the SIR. To improve the SIR to be above 0 dB, the order of the uplink filter is about four-orders according to Equation (6) provided in [22], supposing that the uplink filter is based on the Butterworth filter.

(5) $$\gamma_{\mathrm{SIR}}=20\log\left(\frac{V_{d}\left(j\omega_{\mathrm{ul}}\right)}{V_{d}\left(j\omega_{\mathrm{dl}}\right)}\right),$$

(6) n=log10−0.1γSIR−1logωdlωdlωulωul.

## 4. Minimizing Interactions between NFC and WPT

In this section, we will analyze the two types of interactions. One is the interactions between the NFC uplink and WPT, and the other is the interactions between the NFC downlink and WPT.

### 4.1. Minimizing Interactions between NFC Uplink and Power Link

The uplink and the power link share the same inductive link using different carriers. Thus, they suffer strong interactions as follows:

(1) The strong power carrier will overwhelm the uplink signal and saturate the uplink filter in the out-body receiver.
(2) The RC divider, which is used to avoid saturating the uplink filter, introduces extra thermal noises and degrades the received uplink signal.
(3) The PTE of the power link decreases.

**Figure 4:**
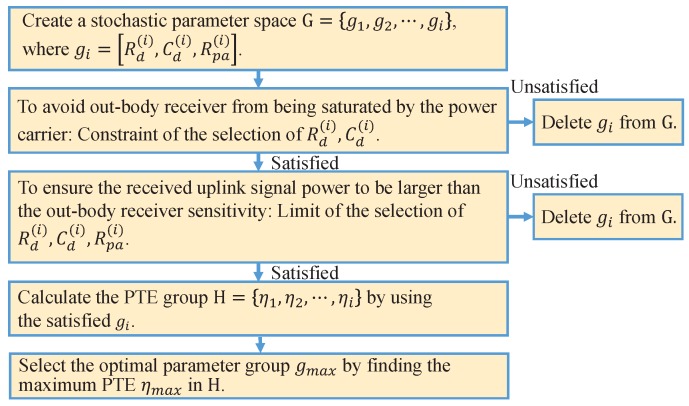
Optimization procedure for the inductive link circuit parameters.

In the following, we firstly explore the circuit parameters that satisfy the constraint of avoiding saturation and keep the received uplink signal power to be sufficient for reliable demodulation. Then, we select the optimal circuit parameters to get the maximal PTE. An overview of the optimization process is shown in Figure 4. We get the approximate global optimal solution in MATLAB by utilizing the Monte Carlo method. The details of each optimizing step are described in the following subsections.

#### 4.1.1. Avoiding the Out-Body Receiver from Being Saturated by the Power Carrier

In the out-body receiver, we introduce the RC voltage divider to prevent the uplink filter from being saturated. The divide ratio of the RC divider is calculated according to the ratio between the power carrier voltage on $L_{o}$ and the maximal input voltage limit of the uplink filter. Since the PDL ($P_{\mathrm{load}}$) should be kept constant to about 5 mW (the total power requirement of our device for the future integrate circuit (IC) design), the power carrier amplitude on $R_{\mathrm{load}}$ can be derived in Equation (7), where $\omega_{\mathrm{dl}}=2\pi \times 2$ MHz is the angular frequency of the power carrier. By using Equation (7) and the transfer function in Equation (3), $V_{o}\left(j\omega_{\mathrm{dl}}\right)$ (the resonated power carrier amplitude on $L_{o}$) can be derived. Through calculating, $V_{o}\left(j\omega_{\mathrm{dl}}\right)$ could vary from 220 V to 22 V in the operating range (0.01<k<0.1).

(7) $$V_{\mathrm{load}}\left(j\omega_{\mathrm{dl}}\right)=\sqrt{2P_{\mathrm{load}}R_{\mathrm{load}}}\approx 4.5\;V,$$

The maximal input voltage allowed for the uplink receiving filter without being saturated is 2 V. Thus, the divide ratio of the RC voltage divider is required to fulfill Equation (8), where ABS() is a function to calculate the absolute value. After simplifying Equation (8), we can derive the constraint of $R_{d}$ and $C_{d}$, which is shown in Equation (9).

(8) $$\mathrm{ABS}\left(\frac{1}{1+j\omega_{\mathrm{dl}}R_{d}C_{d}}\right)\leq \frac{2}{220},$$

(9) $$R_{d}C_{d}\geq 8.8\times 10^{-6}\;\Omega F.$$

#### 4.1.2. Calculating the Sensitivity Limit of the Out-Body Receiver

**Figure 5:**
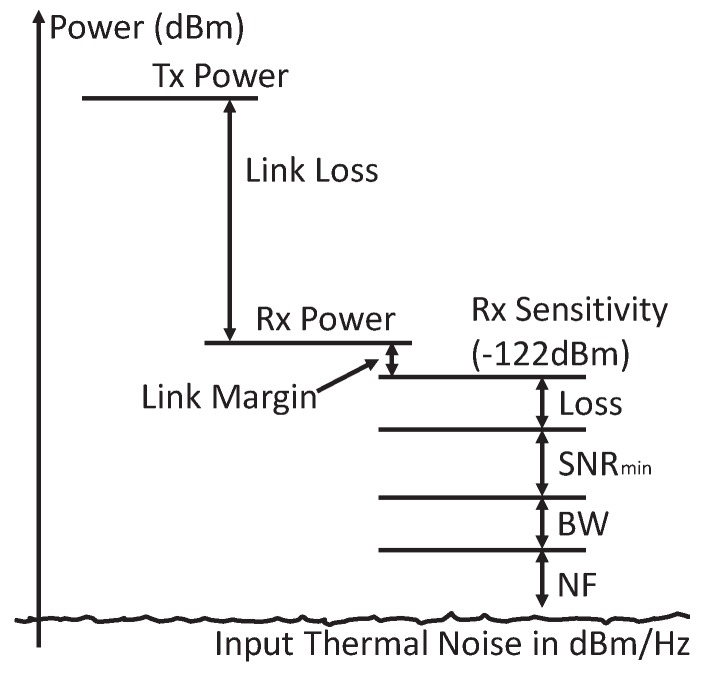
The link budget analysis of the uplink.

Except for the power carrier interference, the thermal noise is another challenge for demodulating the weak uplink signal. To demodulate the uplink data successfully, the minimum received uplink signal power is required to be higher than the sensitivity of the out-body receiver. The sensitivity is derived in Equation (10), where $N_{0}$ is the input noise spectrum density in dBm/Hz; BW is the bandwidth; ${\mathrm{SNR}}_{\min}$ is the minimal required signal-to-noise ratio (SNR) for the reliable demodulation; NF is the receiver circuit noise figure in dB; loss is the receiver circuit implementation loss in dB [23]. Figure 5 shows the complete link budget analysis of the uplink.

(10) $$\mathrm{Sensitivity}=N_{0}+10\log\left(\mathrm{BW}\right)+{\mathrm{SNR}}_{\min}+\mathrm{NF}+\mathrm{Loss}.$$

The input noise of the out-body receiver is the average thermal noise (Johnson noise) on $C_{d}$ of the RC divider. Thus, $R_{d}$ and $C_{d}$ determine the input noise spectrum density $N_{0}$. The $N_{0}$ on $C_{d}$ at $f_{\mathrm{ul}}$ is derived in Equation (11), where $k_{B}$ is Boltzmann’s constant in joules per Kelvin; *T* is the resistor’s absolute temperature in Kelvin; $\omega_{\mathrm{ul}}$ is the angular frequency of the uplink carrier. According to our circuit parameters, the input noise spectrum density $N_{0}$ is −178.5 dBm/Hz, which is better than the Johnson noise floor (−174 dBm/Hz) on resistors because of the $C_{d}$, when *T* = 300 K.

(11) $$N_{0}=10\log\left(4k_{B}T\frac{\omega_{\mathrm{ul}}R_{d}C_{d}}{1+{\left(\omega_{\mathrm{ul}}R_{d}C_{d}\right)}^{2}}\right).$$

The ${\mathrm{SNR}}_{\min}$ in Equation (10) is calculated in Equation (12), where $E_{b}/N_{0}$ is the ratio of energy-per-bit over noise spectral density, and DR is the data rate. For BPSK, the bandwidth approximates the data rate. Thus, the SNR approximates $E_{b}/N_{0}$. The minimum required $E_{b}/N_{0}$ could be estimated by the probability of error. The work in [23] gives the equation that presents the relation between the probability of error and $E_{b}/N_{0}$. If the probability of error is required to be less than $1\times 10^{-7}$, $E_{b}/N_{0}$ should be more than 11.5 dB for BPSK. That is, the ${\mathrm{SNR}}_{\min}$ is 11.5 dB in our system.

(12) $$\mathrm{SNR}=\frac{E_{b}}{N_{0}}\cdot \frac{\mathrm{DR}}{\mathrm{BW}}.$$

The noise figure and the implementation loss are about 3 dB and 2 dB separately in our system. The low implementation loss benefits from the direct demodulation on the samples of the uplink carrier, which is described in Section 5.2. Consequently, the sensitivity of the uplink receiving is −178.5+40+11.5+3+2=−122.0 dBm in our system. The received uplink signal power is required to be larger than −122.0 dBm to achieve reliable uplink demodulation.

#### 4.1.3. Selecting the Optimal $R_{d}$ and $C_{d}$

$R_{d}$ and $C_{d}$ are optimized to achieve the highest PTE. At the same time, the selection of $R_{d}$ and $C_{d}$ should prevent the uplink receiving filter from being saturated and supply sufficient received uplink signal power for the demodulation. Equation (9) provides the constraint to prevent the uplink filter from being saturated.

The PTE η is the ratio between $P_{\mathrm{load}}$ (the power delivered to $R_{\mathrm{load}}$) and $P_{\mathrm{dl}}$ (the transmitted power of the source $V_{\mathrm{dl}}$). It is calculated in Equation (13), where VloadjωdlVloadjωdlVdljωdlVdljωdl and Ii1jωdlIi1jωdlIo1jωdlIo1jωdl are given in Equations (1) and (2).

(13) $$\eta =\frac{P_{\mathrm{load}}}{P_{\mathrm{dl}}}=\frac{V_{\mathrm{load}}\left(j\omega_{\mathrm{dl}}\right)I_{i1}\left(j\omega_{\mathrm{dl}}\right)}{V_{\mathrm{dl}}\left(j\omega_{\mathrm{dl}}\right)I_{o1}\left(j\omega_{\mathrm{dl}}\right)}.$$

The received uplink signal power equals the power of the uplink carrier on $C_{d}$, because the input impedance of the out-body receiver is much higher than that of $C_{d}$. The received uplink signal power could be calculated in Equation (14), where $V_{d}\left(j\omega_{\mathrm{ul}}\right)$ is the uplink carrier amplitude on $C_{d}$. The $V_{d}\left(j\omega_{\mathrm{ul}}\right)$ can be calculated by using the output voltage ($V_{\mathrm{ul}}=3.3$ V) of the in-body uplink PA and the uplink transfer function of $\frac{V_{d}\left(s\right)}{V_{\mathrm{ul}}\left(s\right)}$ in Equation (4).

(14) $$P_{d}\left(j\omega_{\mathrm{ul}}\right)=\mathrm{ABS}\left(\frac{1}{2}{V_{d}}^{2}\left(j\omega_{\mathrm{ul}}\right)\omega_{\mathrm{ul}}C_{d}\right),$$

**Figure 6:**
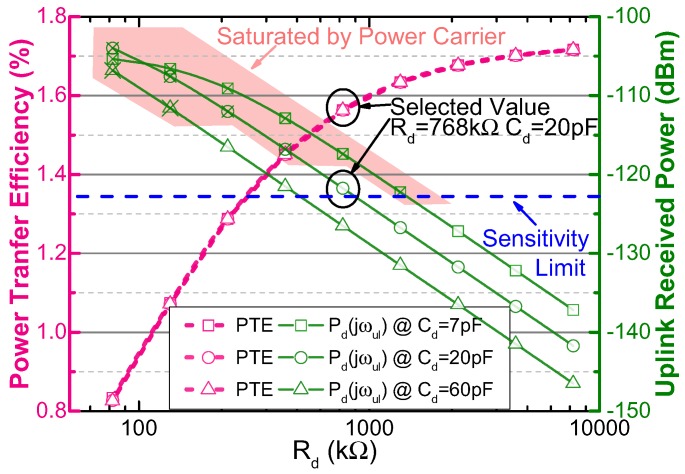
Calculated PTE and the received uplink signal power at $V_{d}$ versus $R_{d}$ and $C_{d}$, when k=0.01.

Figure 6 shows the PTE and the received uplink signal power versus $R_{d}$ and $C_{d}$, when k=0.01, which is the lowest value in the operating range. When the value of *k* is lowest, the received uplink signal power is smallest, and the power carrier interference is strongest due to the adaptive power adjustment mentioned in Section 2.1. That is, the uplink SIR is the lowest in this situation, which is the worst case for the uplink communication. If the uplink could communicate when *k* is the lowest value in the operating range, the uplink can be reliable in the whole operating range. Figure 6 denotes that the change of $R_{d}$ affects both the PTE and the uplink received power, while $C_{d}$ only affects the uplink. The value of $C_{d}$ is selected to get higher uplink received power, as reducing $C_{d}$ could improve the uplink received power. However, too small $C_{d}$ will violate the constraint of avoiding the uplink receiving filter from being saturated (in Equation (9)). The groups of $C_{d}$ and $R_{d}$ that violate the constraint are enclosed by the pink polygon in Figure 6. In order to obtain a high uplink received power without saturating the uplink filter, $C_{d}$ is selected as 20 pF. When $R_{d}$ becomes larger, the PTE increases while the uplink received power decreases remarkably, because the RC divider also scales down the uplink carrier. $R_{d}$ is selected to be 768 kΩ because it benefits the higher PTE, and at the same time, it makes the received uplink signal power always higher than the sensitivity limit of the out-body receiver in the operating range.

#### 4.1.4. Selecting the Optimal $R_{\mathrm{pa}+}$ and $R_{\mathrm{pa}-}$

**Figure 7:**
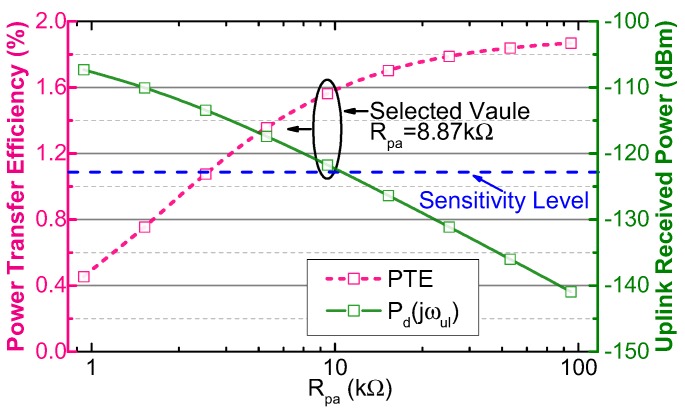
Calculated PTE and received uplink signal power versus $R_{\mathrm{pa}}$, when k=0.01.

To analyze concisely, without losing generality, the values of $R_{\mathrm{pa}+}$ and $R_{\mathrm{pa}-}$ are set to be equal and denoted as $R_{\mathrm{pa}}$. Figure 7 shows the PTE and the uplink received power versus $R_{\mathrm{pa}}$. In order to achieve higher PTE while ensuring that the uplink received power is above the sensitivity limit of the out-body receiver, $R_{\mathrm{pa}}$ is selected to be 8.87 kΩ.

$R_{\mathrm{pa}}$ is also a key parameter of the uplink transmitted power, because it dominates the load impedance of the uplink PA. Thus, the uplink transmitted power is approximately calculated by Equation (15). When $R_{\mathrm{pa}}=8.87$ kΩ, the uplink transmitted power is about 0.61 mW (−4.3 dBm) and only takes 10.8% of the required PDL (5 mW).

(15) $$P_{\mathrm{Tx},\mathrm{ul}}\approx \frac{{V_{\mathrm{ul}}}^{2}}{2R_{\mathrm{pa}}}.$$

### 4.2. Minimizing the Impact of the NFC Downlink on the Power Link

When the downlink data are modulated on the power carrier, the modulation could induce serious amplitude variation on the power carrier. We use BPSK for the downlink, because it theoretically has a constant carrier amplitude and a constant carrier frequency, thus bringing less amplitude variation on the power carrier compared to ASK and less link gain variation on the resonated inductive link compared to FSK.

**Figure 8:**
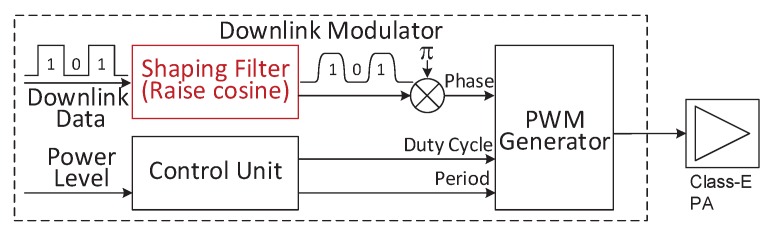
The block diagram of the out-body downlink modulator and a shaping filter is introduced to reduce the phase jump impact of BPSK on Class E PA.

**Figure 9:**
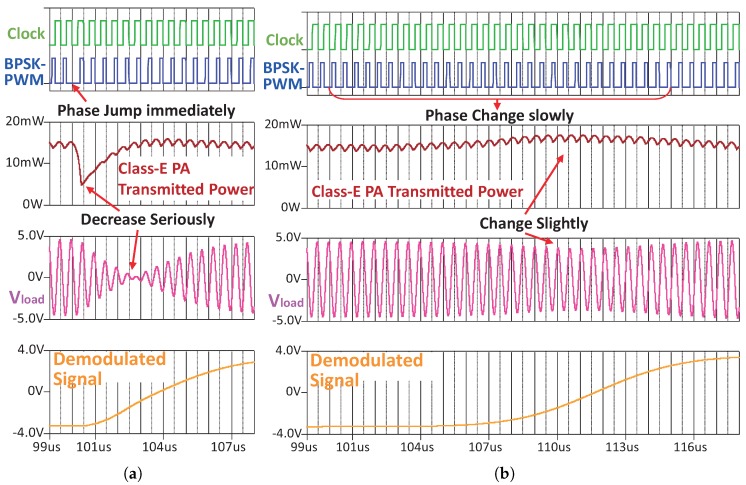
Downlink waves recorded from PSpice simulation, when the BPSK is modulated on the power link (**a**) without using the shaping filter and (**b**) with using the shaping filter.

However, BPSK modulation could generate a phase jump on the power carrier. The phase jump will seriously reduce the transmitting power of the Class E PA, because the Class E PA is very sensitive to its optimal condition (narrow bandwidth). In order to reduce this impact, we introduce a shaping filter (raise-cosine finite-impulse-response digital filter) in the out-body downlink modulator, as seen in Figure 8. The shaping filter smooths the downlink baseband signal. Then, the smooth baseband signal controls the phase of the PWM generator to generate a PWM with smooth BPSK modulation. By feeding the Class E PA, the PWM with smooth BPSK modulation, the transmitting power of the Class E PA will not decrease seriously. Figure 9a shows the impact of phase jump without shaping filter. By using the shaping filter, the phase shift is accomplished slowly in multiple power carrier periods. The phase jump thus is avoided, and then, the serious variations of the Class E transmitted power and $V_{\mathrm{load}}$ (the voltage on in-body coil) are eliminated, as seen in the waves in Figure 9b.

## 5. Experimental Results

### 5.1. Prototype

**Figure 10:**
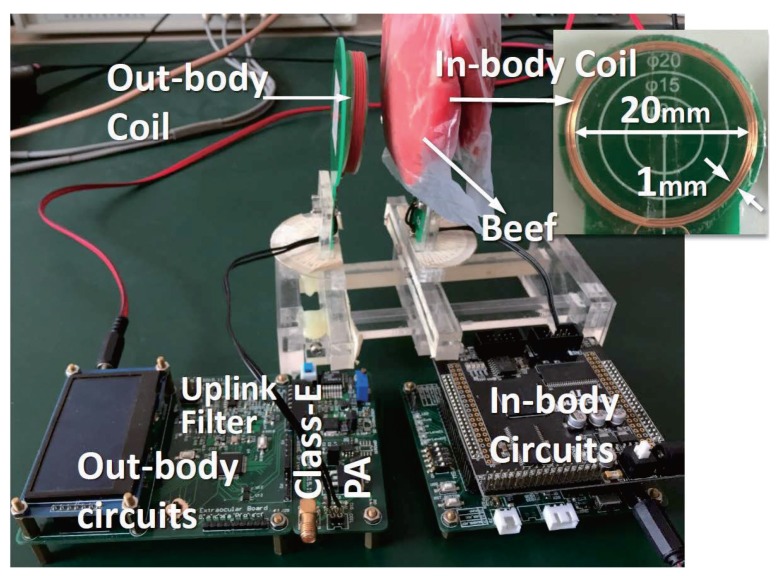
The prototype system was implemented to setup the measurements of power and bi-directional data transmissions with beef tissue.

We implemented our prototype using discrete components and off-the-shelf chips to prove the feasibility of the related IC design, as shown in Figure 10. The circuit parameters in the prototype are the same as the parameters optimized in Section 4. The out-body digital circuits (including the downlink modulator, uplink demodulator and the out-body controller) were implemented in an MCU (STM32F207, STMicroelectronics, Geneva, Switzerland). The in-body digital circuits (including the uplink modulator and the downlink demodulator) were implemented in an FPGA (Cyclone IV, Altera, San Jose, CA, USA).

The overall power consumption of the prototype (including out-body and in-body circuits) is 63.8 mW when the coil separation is 20 mm, and when the system is on standby, the overall power is about 1 mW. The average period of a complete transaction including IOP sensing and communication is about 0.05 s. If the monitoring frequency is once every 5 s and the out-body battery is a small lithium-ion cell with the capacity of 0.5 Wh, the estimated battery life would be about 12.8 days, which is acceptable to the patient to recharge the glasses every 12 days.

**Figure 11:**
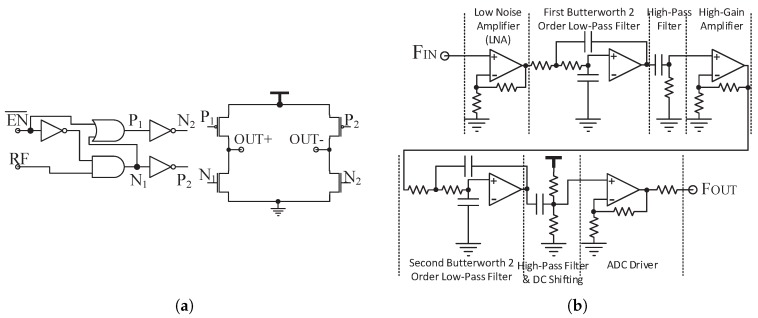
(**a**) The circuit diagram of the in-body uplink PA; and (**b**) the circuit diagram of the out-body uplink filter [18].

The uplink PA circuit of the in-body transmitter and the uplink filter circuit of the out-body receiver are shown in Figure 11. They have been presented in our conference paper [18]. The uplink PA is merged in an H-bridge modulator to achieve low power and high efficiency as seen in Figure 11a. The uplink filter consists of a low noise amplifier (LNA), two Butterworth active filters, two high-pass passive filters and a driver of the analog to digital converter (ADC). The two Butterworth filters are set to be the two-order low-pass filter to block the high frequency power carrier interference. The high-pass passive filters are used to cancel the DC offset of amplifiers and to reject pink noise. The LNA of the uplink filter circuit uses an operational amplifier (OPA656, Texas Instruments, Dallas, TX, USA) with low noise, high input impedance and wide bandwidth.

Since the prototype was implemented by the discrete components, it could consume much more power than that to be implemented in the integrated circuit (IC). Because the low power IC is not yet implemented, the delivered power from the WPT was not sufficient to supply the in-body circuits of the prototype. However, in order to ensure the power supply condition to be well consistent with that of the practical situation, the in-body uplink PA and the in-body downlink comparator were powered by the WPT.

### 5.2. Measurement Results

**Figure 12:**
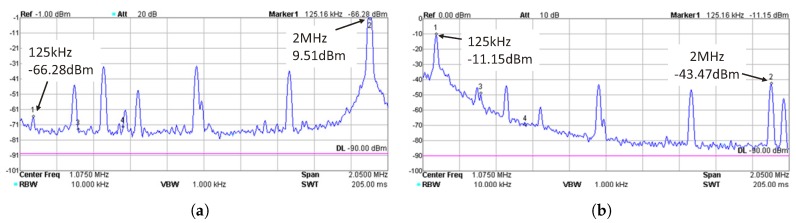
(**a**) The spectrum of the uplink received signal before the uplink receiving filter; and (**b**) the spectrum of the uplink received signal after the uplink receiving filter.

Figure 12a,b shows the measured spectrum of the input and output signals of the uplink receiving filter, respectively, when the coil separation *d* is 20 mm. Figure 12a is measured at the output of the LNA in the uplink receiving filter. The measured results are measured using a spectrum analyzer (DSA815, RIGOL, Beijing, China). By comparing Figure 12b with Figure 12a, we can see that the received uplink signal power is improved from −66.28 dBm to −11.15 dBm. The power of the power carrier interference is suppressed from 9.51 dBm to −43.47 dBm. Thus, the uplink filter improves the uplink SIR from −75.79 dB to 32.32 dB, which is about 108 dB. As seen, there are several peaks between 125 kHz and 2 MHz. They are the noise induced by the DC-DC convertors in the prototype. The noise has negligible impact on the uplink demodulation, since its power is much lower than the received uplink signal power.

**Figure 13:**
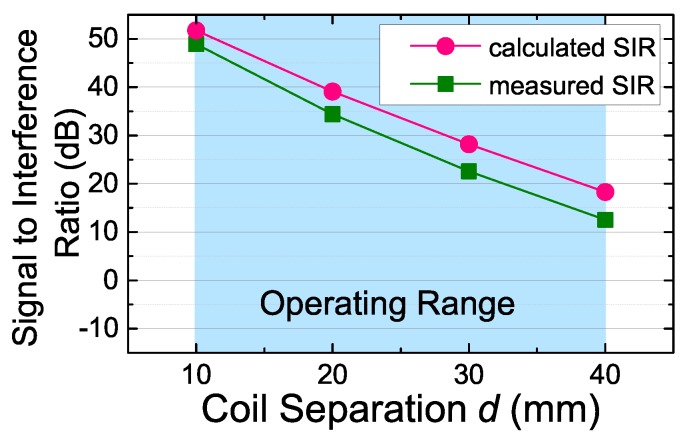
Measured and calculated uplink SIR at the output of the uplink filter versus the coil separation *d*.

We measured the SIR after the uplink receiving filter at different coil separations. Figure 13 shows the measured and calculated uplink SIR versus the coil separations. As seen, when the coil separation is the maximum (40 mm) in the operating range, the SIR is the minimum. The value of the minimum SIR is above 13 dB, which is sufficient for demodulating. In addition, there is only a 5-dB offset between the measured SIR and the calculated SIR, which implies that our circuit modeling is consistent with the practical situation of the prototype.

**Figure 14:**
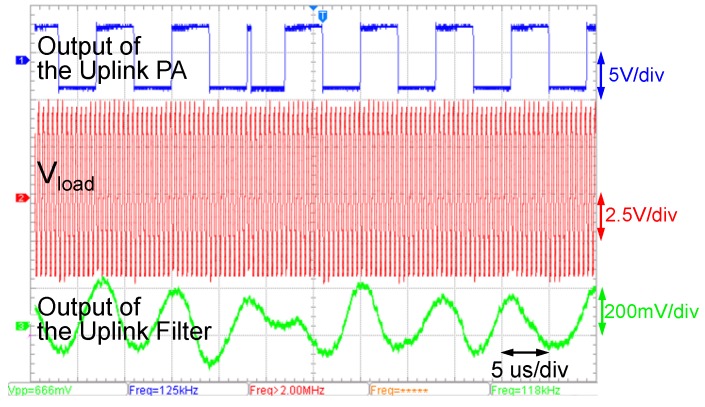
Waveforms in our prototype when the coil separation is 20 mm.

Figure 14 shows the waveforms of three captured signals, which are the output of the uplink PA in the in-body transmitter (not yet merged into the power carrier), the $V_{\mathrm{load}}$ (the power carrier waveform on the in-body coil) and the output of the uplink receiving filter in the out-body receiver. The output of the uplink PA is a BPSK-modulated square wave signal. The $V_{\mathrm{load}}$ estimates the ripples of $V_{\mathrm{load}}$ induced by the in-body uplink PA. The uplink modulation caused negligible ripple (about ∼5% according to the oscilloscope download data) on $V_{\mathrm{load}}$. Through the filtering and the amplifying, the uplink signal can be clearly visible, and the modulated phase inverting is significant, while the interference of the power carrier is sufficiently suppressed by the uplink receiving filter.

**Figure 15:**
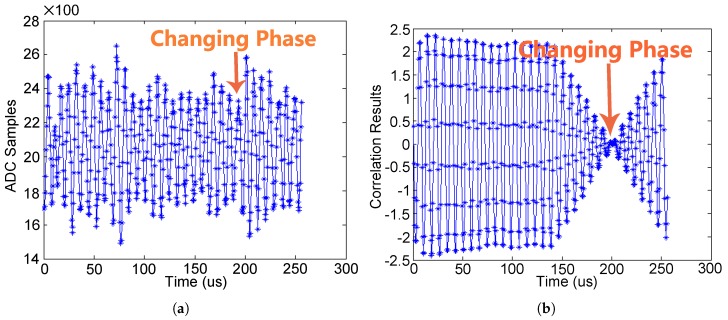
(**a**) ADC samples of the uplink receiving filter output; and (**b**) the correlation results of the matched filter in the uplink demodulator.

Direct ADC sampling on the uplink carrier (125 kHz) is applied, and the match-filter for demodulation is directly performed right after ADC. The match-filter cross-correlates the 125-kHz local sinusoidal signal with the ADC output. Figure 15a shows the ADC output. As seen, there exists parasitic amplitude modulations, and it is hard to detect the phase changing directly from the uplink carrier. Figure 15b shows the correlation results of the matched filter. As seen, after the matched filtering, the phase changing is exposed clearly, as shown in Figure 15b.

**Figure 16:**
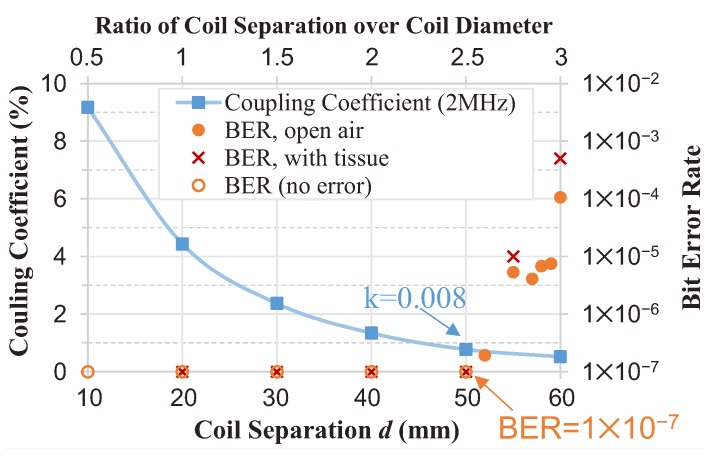
The measured uplink BER and the coupling coefficient versus the coil separation, when the PDL is kept constant at 5 mW.

Figure 16 shows the measured results of the uplink BER and the coupling coefficient over different coil separations. As seen, when the coil separation is within the operating range, the BER of the uplink can be down to zero in half an hour. When the separation is 50 mm (k=0.008), the bit error is measured. The large distance demonstrates the capacity of reliable data transmission under ultra-weak coupling. To examine the effect of tissue on the data links, the BER was measured with beef tissue (of a thickness of 10 mm) tightly covering the in-body coil, as shown in Figure 10. The measured BER of the uplink with the beef is also plotted in Figure 16. The results show that the tissue induces a negligible impact on the BER.

The coupling coefficient and the PTE of the inductive link are measured at 2 MHz by using a network analyzer (E5071, Keysignt, Santa Rosa, CA, USA) through the method introduced in [24,25]. When measuring the PTE, a 2 kΩ resistor is substituted as the load.

To measure the BER, a pseudo-random data sequence is transmitted continuously at a speed of 10 kbps in half an hour for each measurement at different coil separations. The uplink and the downlink are measured separately, as they work in half-duplex. When measuring the BER, the PDL is kept as constant as 5 mW by adjusting the transmitted power of the Class E PA.

To keep the PDL constant, the transmitted power of the Class E PA is adjusted. Thus, the power carrier interference is stronger when the distance is large. The uplink filter of the out-body receiver was saturated by the power carrier when *d* approached 70 mm. However, as the transmitted power of the power carrier is increased according to the adaptive power adjustment, the downlink BER keeps below $10^{-7}$ within our operating range.

**Figure 17:**
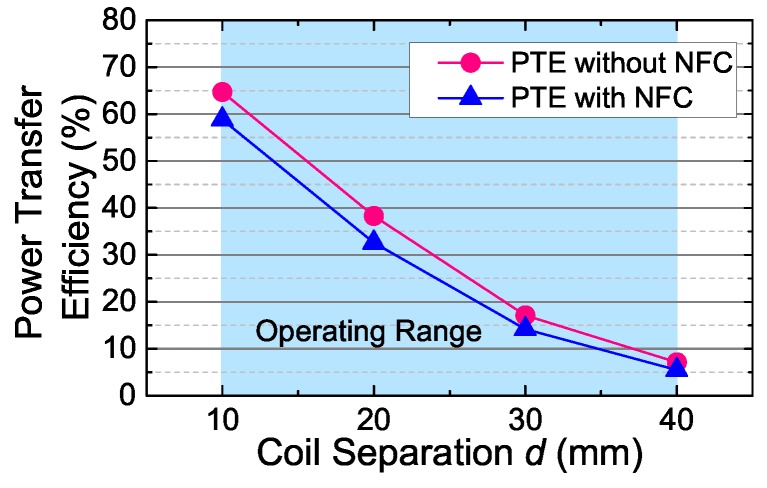
The power transfer efficiency of WPT versus coil separation *d* with or without connecting the added NFC circuits.

We measured the PTE with and without the NFC circuits (disconnect and disable the transceivers) separately to estimate the PTE loss caused by the NFC circuits. The measured results are listed in Figure 17. As seen, although the NFC shares the same coil pair with the WPT, the total PTE still maintains a sufficiently acceptable level without significant drop.

**Figure 18:**
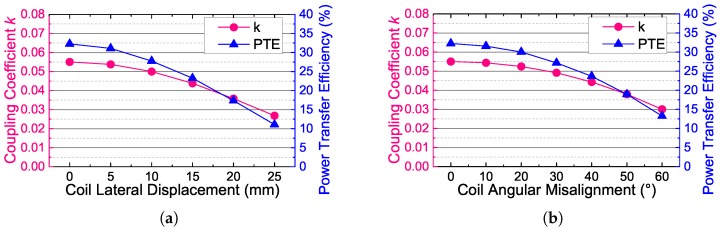
The coupling coefficient and the power transfer efficiency versus (**a**) coil lateral misalignments and (**b**) coil angular misalignments when the coil separation d=20 mm.

The coupling coefficient and the power transfer efficiency are measured over different coil misalignments to evaluate the effects of eyeball movements and position displacement of glasses. We classify the coil misalignments into two types. One is the lateral displacement, which corresponds to the changes of the glasses position, and the other is the angular misalignment, which corresponds to the eyeball turning. The measured results are shown in Figure 18. As seen, the coupling coefficient and the PTE are not very sensitive to the coil misalignments. This is benefited from the simple coil configuration and the large size of the out-body coil of our system. According to the statistics from our cooperative hospital, the normal turning of human eyeball is within ±30${}^{\circ }$, and the normal lateral displacement of the glasses is within 10 mm. Therefore, the normal turning of human eyeball could lead to about an 11% decrease of the coupling coefficient and a 16% decrease of the PTE, and the normal lateral displacement of the glasses could lead to about a 10% decrease of the coupling coefficient and a 14% decrease of the PTE. These effects of the coil misalignments are acceptable to the wireless power transfer and near-field communication of our system.

### 5.3. Tissue and Safety

**Figure 19:**
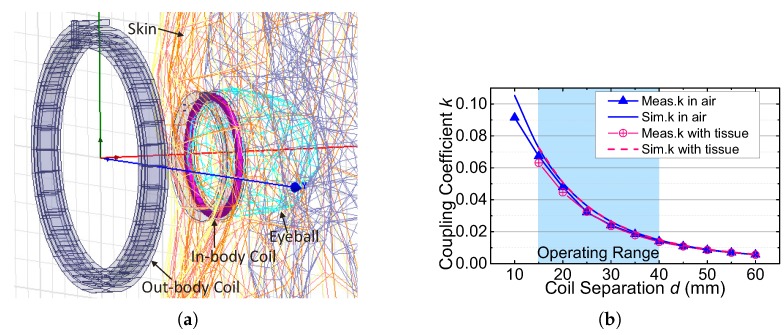
(**a**) Inductive link simulation with the human head model in ANSYS HFSS; and (**b**) measured and simulated *k* in air and in tissue.

The in-body coil $L_{i}$ is sealed in silica gel and placed in tissue. The tissue increases its parasitic capacitance and decreases its self-resonated frequency and quality factor [26]. However, benefiting from the low frequency of the uplink carrier, the impact of the tissue is minor in our wireless link. As shown in Figure 16, the beef tissue induces negligible impact on the BER. According to the work in [27], the permittivity and the conductivity of the beef muscle tissue is close to those of the human muscle tissue when the frequency is about 2 MHz. As our in-body coil will be implanted around the eyeball, the tissues in the eyeball, especially the vitreous humor, need to be further studied. We simulate our inductive link where the in-body coil is sealed in silica gel with the detailed true-CAD human model in ANSYS HFSS [28], as shown in Figure 19a. The human model contains the tissues of skin, fat, muscle, eyeball and skull. The dielectric properties of the tissues are derived from [29]. Figure 19b shows the measured coupling coefficients when the in-body coil is in air or is covered by the beef tissue and the simulated coupling coefficients when the in-body coil is in air or is placed in the human body model over different coil separations. Figure 19b presents that the coupling coefficients with tissue are almost the same as the coupling coefficients measured/simulated in air. The tissues have negligible impacts on the coupling coefficients, because the low frequency magnetic field can pass through the tissues with little loss.

Specific absorption rate (SAR) is an important metric to evaluate the radio frequency (RF) exposure safety on the human body. The ANSYS HFSS electromagnetic simulation suite was used to study the SAR. In the simulation results, the maximal local SAR in tissue is 1.7 × 10${}^{-3}$ W/kg in 10 g in our operating range. It is about three orders lower than the basic restriction 2 W/kg [30].

In addition, power dissipated by the in-body coil and in-body circuits could also lead to the temperature increase in the human body. According to the comprehensive thermal analysis on an intraocular implant [31], our device with 5 mW power consumption may induce the maximum temperature increase of approximately 0.4 ${}^{\circ }$C on the surface of the device. The temperature increase is below the guideline 1 ${}^{\circ }$C in [30].

### 5.4. Comparison with the State-of-the-Art

A comparison of the proposed NFC with other published works is shown in Table 2. We carefully select reference papers that focus on power transmission and communication on the same inductive link. As in this case, the performance of the data link is limited by the power link. As seen in Table 2, our work novelly uses dual carriers and BPSK modulation for both downlink and uplink to achieve reliable communication. Through optimizing the inductive link circuit parameters, the proposed NFC achieves reliable communication (BER < 1× 10${}^{-7}$) when the coupling coefficient is five- to 10-times lower than the minimal coupling coefficient achieved by other referenced works. At the same time, the proposed NFC coexists better with the WPT. The proposed NFC not only overcomes the strong interference (−100 dB SIR) of the power carrier, but also induces less PTE loss and smaller voltage ripples on the power link compared with other referenced works.

The proposed dual-carrier NFC is able to work under the ultra-weak coupling. In this paper, the target application requires the coupling coefficient to be as low as 0.01. Moreover, we optimize the same dual-carrier system to reliably transmit uplink data under the coupling coefficient as low as 0.003 with a data rate of 2 kbps in [18]. Although the coil size of our prototype may be larger than the coil size of other works, the large coil separation could lead to the same ultra-weak coupling situation. Therefore, this NFC system could be an available option for the data transmission to the recent mm-sized implantable biomedical sensor systems [2].

## 6. Conclusions

A dual-carrier NFC, attached to an inductive WPT link, has been proposed and optimized for simultaneously transmitting power and bi-directional data via the same ultra-weak coupled coil pair. To demodulate the weak uplink signal under strong power carrier interference, a dedicated circuit is designed and introduced to the inductive link. The parameters of the dedicated circuit are optimized to minimize the interactions between the NFC and the WPT. We use our implantable sensor system for glaucoma treatment as an application example to present the dual-carrier NFC system and to elaborate its optimization procedure. The prototype achieves reliable (BER = 1 × 10${}^{-7}$) communication even though the coupling coefficient is as low as 0.008, and the NFC coexists well with the WPT with only 15% PTE loss and ∼5% voltage ripple. The proposed NFC is very suitable for the battery-less implantable biomedical sensor systems with strict limits on coil size and coil number. The proposed NFC scheme and its optimization procedure could also be useful for other small-sized implantable biomedical sensor applications.

## Figures and Tables

**Table 1:** Parameters of The Coils.

Parameter	$L_{o}$	$L_{i}$
Coil Wire Type	Litz wire with 11 strands of 30 AWG	Enameled copper
Wire Diameter	0.8 mm	0.06 mm
Coil Diameter	40 mm	20 mm
Coil Thickness	4.5 mm	0.12 mm
Number of Turns	30	35
Coil Inductance	68.2 μH ^a^	65.5 μH ^a^
Equivalent Series Resistance	8.5 Ω ^a^	21.2 Ω ^a^
Quality Factor	100.8 ^a^	38.8 ^a,b^

${}^{a}$ Measured by a precision impedance analyzer (Keysignt E4990A) at the power carrier frequency; ${}^{b}$ the low quality factor of the in-body coil is due to the high resistance of the thin wire.

**Table 2:** Comparison with Other Single-inductive-link Designs.

References	[32]	[33]	[34]	[35]	This Work
Coil Number	2	2	3	2	2
Coil Diameter (mm)	60/20	40/22	35/12	25/16	40/20
**Wireless Power Transfer (Power Link)**					
Carrier Frequency	700 kHz	1 MHz	13.56 MHz	13.56 MHz	2 MHz
Typical PDL (mW)	50	250	>2.3	≤100	5
Overall Power (mW)	137.3	689	NA	NA	63.8
Typical PTE	51%	38%	NA	58% (k = 0.1)	59% (k = 0.1)
**Near-Field Communication (Downlink/Uplink)**					
Carrier Frequency	700 kHz/-	1 MHz/-	13.56 MHz/-	13.56 MHz/-	2 M/125 kHz
Data Rate (bps)	60 k/-	3 k/-	100 k/-	400 k/1.35 M	10 k
Modulation	ASK/LSK	-/LSK	BPSK/LSK	OOK/PPSK	**BPSK/BPSK**
Duplex Type	Half-duplex	Uplink	Full-duplex	Half-duplex	Half-duplex
Distance (mm)	30	15	10	15	**50**
Coupling Coefficient	0.045	0.08	0.075	0.055	**0.008**
BER	NA	≤1.6 × 10${}^{-6}$ (PER)	NA	≤1 × 10${}^{-5}$	**≤1 × 10${}^{-7}$**
**The Impacts of NFC on WPT**					
PTE Relative Decrease	50%	NA	NA	20% (k = 0.05)	**15% (k = 0.05)**
$V_{\mathrm{load}}$ Ripple	NA	NA	NA	100%	**∼5%**

${}^{a}$ They used two coils at the implant side to prevent the LSK transmitter from interfering with the inbound BPSK data signal transfer and power transfer. ${}^{b}$ The 5-mW PDL is our system requirement. The 10 Kbps data rate is sufficient for our implantable sensor system for glaucoma treatment. ${}^{c}$ The PTE is the power transfer efficiency of the coupling link excluding the efficiency of the power amplifier and the efficiency of the rectifier. ${}^{d}$ OOK(On-off keying); PPSK (Passive phase shift keying). ${}^{e}$ Simulated by using ANSYS HFSS from the coil parameters. ${}^{f}$ Equivalent to a 4 × 10${}^{-5}$ packet error ratio (our packet size is 24 bits).

## Abbreviations

The following abbreviations are used in this paper:

AC	Alternating current
ADC	Analog to digital converter
ASK	Amplitude shift keying
BER	Bit error rate
BPSK	Binary phase shift keying
BW	Bandwidth
DC	Direct current
DR	Data rate
DPSK	Differential phase shift keying
FSK	Frequency shift keying
HIPR	High impedance probe resistor
IOP	Intraocular pressure
LNA	Low noise amplifier
LSK	Load-shift keying
NF	Noise figure
NFC	Near-field communication
OQPSK	Offset quadrature shift keying
PA	Power amplifier
PDL	Power delivered to load
PTE	Power transfer efficiency
PWM	Pulse-width modulation
RFID	Radio frequency identification device
SAR	Specific absorption rate
SIR	Signal-to-interference ratio
SNR	Signal-to-noise ratio
WPT	Wireless power transfer
